# Self-organizing maps for allele specific expression data reconstruction and identification of anomalous genomic regions

**DOI:** 10.3389/fbinf.2026.1810835

**Published:** 2026-04-14

**Authors:** Roberto Pagliarini, Francesco Nascimben, Alberto Policriti

**Affiliations:** Department of Mathematics, Computer Science, and Physics, University of Udine (Italy), Udne, Italy

**Keywords:** allele specific expression, data recovery, errors identification, self-organizing maps, unsupervised clustering

## Abstract

Allele Specific Expression data quantifies expression variation between the two haplotypes of a diploid individual distinguished by heterozygous sites. Current methodologies of genome-wide sequencing produce large amounts of missing data that may affect statistical inference and bias the outcome of experiments. Machine learning tools could be employed to explore the data and to estimate missing signatures. We present a two-phase procedure based on Self-Organizing Maps (SOMs), an unsupervised clustering technique, to recover missing allele specific expression data from RNA-seq experiments. Specifically, a SOM trained on a complete population P is used to assign a so-called corrupted individual $\hat{p}$ to its most fitting cluster c ; then, a completion rule based on allele frequencies within the subpopulation of $P_{c}\subseteq P$ defined by c is employed to reconstruct $\hat{p}$ . To evaluate our approach, we first apply it to purely artificial datasets, in order to have full control over all experimental conditions. After that, we consider a real population of *Vitis vinifera*, which we also extend by applying a computational framework to generate synthetic individuals from allele expression data. We then introduce two local feature relevance indices in order to assess the influence of specific alleles on the topological placement of corrupted individuals in the SOM structure. Our results, showing promising accuracy in the prediction of missing alleles, suggest that the developed approach could be very useful for recovering incomplete samples in a dataset instead of discarding them, mainly in situations where experiments are challenging.

## 1 Introduction

Allele-Specific Expression (ASE) is a phenomenon in diploid organisms in which the two alleles, maternal and paternal copies,of a gene are transcribed at different levels. It acts as a powerful tool to identify cis-regulatory genetic variants and epigenetic changes that cause expression imbalances. ASE provides an internal control for each cell, minimizing environmental confounders, and is measured using RNA sequencing (RNA-seq) to identify heterozygous sites (Mattevi et al., 2025). Unlike total gene expression, ASE measures the relative abundance of transcripts from one allele versus the other. It is primarily driven by cis-regulatory mutations in enhancers, promoters, or silencers that affect one allele specifically. Standard bulk RNA-seq techniques capture differentially expressed alleles only when higher expression of one parental allele is shared among individual organism cells and is often applied in studies of genetic regulation, where the expression levels of alleles inherited from each parent are measured independently (Demirdjian et al., 2020; Fan et al., 2020; Rao et al., 2021).

A major obstacle in ASE reconstruction from RNA-seq data is reference mapping bias: reads carrying the non-reference allele often map less efficiently to the reference genome, leading to spurious reference-allele over-expression. The WASP framework addresses this issue by re-mapping allele-swapped reads and discarding those whose alignment depends on allele identity, thereby substantially reducing false allele imbalance in downstream ASE analyses (van de Geijn et al., 2015). When high-confidence genotypes are missing or incomplete, genotype uncertainty becomes a major source of error, resulting in false heterozygote calls or apparent monoallelic expression due to allelic dropout. To address this limitation, QuASAR introduced a joint probabilistic framework that simultaneously infers heterozygous genotypes and tests for ASE while explicitly modeling sequencing error and uncertainty. This approach is particularly relevant in RNA-only settings or when genotype calls are noisy (Harvey et al., 2014). Beyond locus-specific imbalance, RNA-seq data can also support the detection of broader genomic abnormalities through two complementary signals: allelic imbalance shifts across expressed heterozygous loci, and coordinated regional expression shifts along genomic coordinates. However, a recurrent theme in the literature is that RNA-seq provides sparse and expression-biased genome coverage, limiting resolution. Consequently, RNA-based approaches are generally more reliable for chromosome- and arm-level events, while focal copy number variations (CNVs) remain more challenging to resolve accurately. eSNP-Karyotyping detects chromosomal aberrations by computing allele-expression ratios at expressed Single-Nucleotide Polymorphisms (SNPs) and scanning for regional deviations consistent with copy gains or losses. It works without requiring a matched diploid DNA control sample, making it particularly useful when such controls are unavailable (Weissbein et al., 2016). CaSpER extends this concept by explicitly integrating multiscale-smoothed gene expression profiles with an allelic shift signal derived from RNA-seq reads. Through hidden Markov model based segmentation across smoothing scales, it requires concordance between expression and allelic evidence, thereby addressing a central limitation of RNA-seq—namely, distinguishing regulatory expression changes from true dosage alterations (Serin Harmanci et al., 2020). More recently, RNAseqCNV was specifically developed for chromosomal and arm-level CNV detection from bulk RNA-seq data. It combines normalized gene-level expression with minor allele fraction profiles derived from SNVs and applies sequential random forest classifiers to improve arm-level calls, particularly in highly aneuploid contexts where global normalization is challenging (Bařinka et al., 2022). Collectively, these approaches highlight both the potential and the intrinsic limitations of RNA-seq–based inference of allelic imbalance and copy number variation.

Although methodological advances have reduced mapping bias, genotype uncertainty, and signal sparsity, accurately distinguishing regulatory effects from genuine genomic dosage alterations remains challenging, particularly at high resolution. Existing CNV-oriented methods explicitly model dosage changes and apply segmentation strategies but they are primarily tailored to large-scale chromosomal or arm-level events. In contrast, the present work does not pursue CNV calling or genomic segmentation. What we propose is a complementary unsupervised framework based on Self-Organizing Maps (SOMs) to reconstruct incomplete ASE profiles and identify loci that introduce geometric distortions within a learned low-dimensional representation. By leveraging topology-preserving clustering and feature-wise contributions to quantization and classification margins, our approach captures internal structural inconsistencies in ASE data without explicitly modeling copy number states. By projecting high-dimensional features—such as allelic imbalance, expression levels, coverage, and quality metrics—onto a low-dimensional topology-preserving grid, SOMs can identify coherent patterns of regional behavior without requiring labeled training data. This property is particularly advantageous in rare-disease and small-cohort settings, where supervised learning approaches may be unstable or infeasible. Furthermore, the topology-preserving nature of SOMs facilitates the detection of anomalous loci or genomic regions exhibiting inconsistent allelic and expression signals, potentially distinguishing biological dosage alterations from technical artifacts. As such, SOM-based modeling may offer a flexible and robust strategy for addressing signal sparsity, genotype uncertainty, and data corruption in RNA-seq–based ASE and CNV inference. In a SOM, a finite set of models is associated to neurons of a regular grid, such that more similar models are closer to each other in the grid (Kohonen, 1982; Kohonen et al., 2001). It is a computational abstraction building on biological models of neural systems and morphogenesis (von der Malsburg, 1973; Turing, 1952). SOMs create internal representations reminiscent of the cortical homunculus based on a neurological map of the areas and proportions of the human brain dedicated to processing sensory functions, for different parts of the body. Specifically, a SOM is trained employing competitive learning rather than error-correction learning (Haykin, 1998). Once the SOM is trained on a dataset, an input data point gets assigned to the neuron (cluster) whose associated model is the most similar to it.

Since SOMs simultaneously perform dimensionality reduction, clustering, and visualization, they are particularly attractive for exploratory analysis and interpretation of complex biological datasets. In (Tamayo et al., 1999), authors show that SOM-based organization of large microarray datasets enables extraction and visualization of dominant expression patterns, facilitating recognition and classification of features in complex, multidimensional gene-expression data. In alignment-free genomics, genome signatures derived from compositional statistics can be projected onto SOMs to support intuitive understanding of relationships across broad phylogenetic scales, with the map acting as an atlas for compositional similarity (Gatherer, 2007). This work also demonstrated that larger SOMs can improve accuracy where classification is possible, but may become less sensitive for classifying unknown sequences and require longer training, motivating smaller/faster maps in many settings. Although SOMs are fundamentally unsupervised, SOM-derived representations within predictive pipelines have been employed. (Fatima and Rueda, 2020) employs SOMs as a mechanism for both dimensionality reduction and interpretable representation learning by integrating them with convolutional neural networks. In microbial and comparative genomics, (Abe et al., 2020) highlights that unsupervised SOM clustering of oligonucleotide composition can support large-scale, alignment-free discovery tasks such as identifying horizontal gene transfer candidates and inferring their likely origins, even under high novelty and limited donor information. Since SOMs utility also depends on input representation, the authors of (Dozono and Niina, 2013) propose a SOM that uses nucleotide correlation-coefficient features, arguing that the input vector can be made substantially smaller while preserving comparable, and sometimes better, clustering clarity across species—directly supporting the efficient but interpretable visualization advantage often attributed to SOMs. A complementary line of research addresses flexibility in clustering and visualization (Ressom et al., 2003): adaptive double SOM approaches are presented as methods that perform clustering and visualization simultaneously by reducing reliance on *a priori* knowledge of the number of clusters. SOMs have also been proposed as tools to organize and visualize genetic diversity patterns in populations (da Silva Oliveira et al., 2020). This study shows that SOMs can map simulated evolutionary effects (drift, selection, migration, inbreeding) from allele/genotype frequencies, reporting that the topological map efficiently organizes populations into patterns consistent with the underlying evolutionary factor.

In this work, we tackle a different biological problem. Specifically, the aim of this manuscript is twofold. Firstly, we tackle a data recovery problem: the goal is to reconstruct a corrupted dataset, i.e., a collection of genomes, also named individuals, on which one or more errors occurred during an RNA-seq experiment, leading to a failure in recognizing some expressed alleles and their associated read-counts: this means that both correctly and incorrectly missing alleles are represented as zeroes in an expression matrix of dimension $\hat{a}\times p$ , where each column is an individual of a population P and each row is an allele, as we shall explain later on. Drawing from a previous work where SOMs were used to perform missing SNP imputation (Mora-Márquez et al., 2025), we develop a system capable of correctly reconstructing corrupted individuals, applying completion rules based on the specific clusters the individuals are classified into by a SOM. Secondly, we address an identification problem by quantifying feature contributions during SOM adaptation: the purpose is the robust recognition of genomics regions with significant and consistent data corruption. The proposed approach is particularly useful when analyzing the impact of corrupted genomes, as it highlights which alleles are responsible for local distortions or shifts in cluster assignment within the SOM topology. SOMs offer several advantages: they are easy to interpret, perform effectively in classification tasks, and allow for straightforward evaluation of their quality. Furthermore, they enable users to intuitively assess both the quality of the resulting maps and the degree of similarity between the analyzed objects. For these reasons, SOMs are particularly well suited for classification applications.

A number of imputation methods have been proposed in the literature. To the best of our knowledge, none of them quite fit the task at hand (recovery of missing expressed alleles and their read counts), due to a combination of these issues: (I1) not being suitable for the high-dimensional regime $\left(\hat{a}\gg p\right)$ ^1^ (Pagliarini et al., 2024); I2) requiring the missing data (allele) to be explicitly marked as N/A in order to predict their expression values, *de facto* bypassing our main detection sub-task; I3) not guaranteeing the biological constraints, e.g., at most 2 expressed alleles per gene, to be respected. Even though we do not claim to be exhaustive, given the vastness of this research field, in the following we cite some of such methods, noting why they are not suitable (or, at least, not without non-trivial pre- or post-data processing) for our task. Random Forests (Breiman, 2001) are a powerful versatile supervised machine learning ensemble method used for classification and regression by training numerous decision trees on random data subsets (bagging) and selecting random feature subsets to minimize correlation and overfitting. Competitive RF-based methods, such as missForest (Stekhoven and Bühlmann, 2012) or ARF (Antonenko et al., 2024), improve on the original concept and might be suitable for the high-dimensional regimes but they are all subject to issue I2. A workaround to this would be to mark every 0-entry in the ASE dataset as N/A but this would yield two problems: first, the dataset would be mostly comprised of N/A entries, compromising the reliability of the imputation; second, each original 0-entry could (and, most likely, would) get a non-zero value, incurring issue I3. MICE, Multiple Imputation with Chained Equations (van Buuren and Groothuis-Oudshoorn, 2011), remains a state of-the-art approach in the imputation domain, but is generally not suited for the $\hat{a}\gg p$ regime (I1) and suffers from issues I2 and I3. kNN-based methods, such as kNNimpute (Troyanskaya et al., 2001), while suitable for large feature-to-population size settings, also require prior identification of deleted alleles. The same holds for matrix factorization (Nguyen et al., 2019; Udell et al., 2016) approaches. Denoising autoencoders, such as (Chen and Shi, 2019), have proved to work well for missing value imputation but they require enough cases for the training phase and cannot guarantee satisfaction of our biological constraints (I3). Other methods, such as diffusion or graph-based imputers, e.g., MAGIC or G2S3 (van Dijk et al., 2018; Wu et al., 2021), both developed for single-cell RNA-seq but theoretically extendable to bulk RNA-seq data, do not require missing values to be explicitly marked, as they perform denoising/dropout imputation on the whole data matrix. However, these methods were developed with (non-allele-specific) gene expression in mind: thus, the resulting denoised matrices would still not be bound to respect the diploid constraint (I3). Overall, while it seems some of the approaches mentioned above, such as MICE, could be adapted by using a different representation of our data (for instance, each individual could be considered as a vector of length 2 g, with g the number of genes, depicting the expressed alleles for each gene as an integer entry, in order to have a single N/A for each missing allele). This would still require careful considerations dictated by the strong, specific constraints of ASE data -assuming to adopt the alternative representation of the previous example, we would need the system, for instance, to be invariant to allele permutation among the two entries reserved for each gene-. These observations, along with the intrinsic simplicity and efficiency of our SOM-based method, which only requires minimum parametrization and can work on raw expression data, make us confident in the validity of our work.

To evaluate our procedure, we use *quality* and *similarity* indices, which assess, respectively, the adherence of the SOM classification to a specific ground-truth labeling and its robustness with respect to the amount of corruption within individuals. Furthermore, we measure the fraction of correctly guessed alleles (*allele accuracy*), among the deleted ones, and the error in read-count estimation. Finally, we also identify those alleles (features) and genes which are most relevant to the displacement of individuals within the SOM structure, using two distinct indices.

As a first case study, to have complete control over all experimental conditions, we show the application of our method to artificially generated datasets, where we observe promising results: the procedure correctly guesses over 90% of missing alleles in test populations, even when up to 30% of each individual’s signature is deleted (see Figure 3). We then apply our approach to a grapevine variants ASE database: in this much more complex case (Figure 4), performance of our indices becomes significantly worse, as expected, further degrading as the amount of corruption increases; however, allele accuracy remains remarkable (between 50 and 60% ), considering the high number of genes involved (1,566) and the variability within them ( ∼10 possible alleles per gene, on average). In order to improve performance, we compute a spectral embedding of the individuals, i.e., an eigenvector-based representation in low-dimensional space, on which we train and evaluate the SOM. This approach (Figure 5) significantly improves similarity and allele accuracy (∼70%) , making all indices extremely robust to corruption. Assuming that the limited size of the population (98 individuals) compared to the feature space (over 16,000 features per individual, most of which are equal to 0) might hinder the learning process of the SOM, we attempt to extend our grapevine dataset by using a synthetic individuals generator we devised for a previous work: the generator, described in detail in Subsection 2.7, takes into input a population and produces a synthetic population which is statistically indistinguishable from the original by employing an integration of evolutionary and genetic algorithms. While overall performance becomes worse ( <50% allele accuracy), results in Figure 6 display a curious behavior for two quality indices, where their values, counterintuitively, improve as the corruption level increases: this was useful to highlight a bias both in the quality indices and in the extended dataset, for which we provide an explanation. For the same extended dataset, we again use the spectral embedding (Figure 7), improving allele accuracy by a margin of ∼5% , confirming the effectiveness of this representation.

As a baseline reference, we also repeat the imputation experiments, replacing SOMs with the standard k -means algorithm for the preliminary clustering phase. Results remain fundamentally identical: we provide a theoretical explanation to this. Finally, on the read-count representation of the real-only dataset, we compute Z-scores, derived from the two relevance indices, to determine which alleles contribute in a statistically significant way to the displacement of individuals in the SOM. We find 22 genes with a significant local error contribution and 10 genes with a significant discriminant, on which we perform enrichment analysis.

These results suggest that our approach may represent a valuable strategy for preserving samples within a dataset rather than discarding them, especially in contexts where experiments are difficult, costly, or simply impossible to repeat. In genomic research, data scarcity is a pervasive limitation—driven not only by the high costs of sequencing and analysis, but also by the intrinsically small number of available subjects, the rarity of many conditions, and the practical and ethical constraints associated with collecting additional biological material. For example, clinical genomic testing in rare diseases can incur substantial expenditures per exome or genome analysed, with sequencing and labour accounting for significant proportions of cost, thereby limiting the scope for repeated or expanded sampling in routine practice (Mordaunt et al., 2024). Moreover, the very nature of certain cohorts makes sample scarcity irreversible: in prospective studies of rare disorders there may simply be too few affected individuals for large-scale data collection, and in retrospective analyses additional specimens may no longer be available. These limitations are well documented in rare disease research, where small sample sizes are often an inevitable consequence of low disease prevalence and have historically impeded statistical power and biological discovery (Mitani and Haneuse, 2020).

In addition to biological rarity and cohort size limitations, RNA-seq–based ASE datasets are particularly sensitive to pre-analytical and storage-related variability. Degradation, uneven preservation, and technical handling differences may introduce missing allelic signals or unreliable read counts, effectively reducing usable sample size (Williams et al., 2014). In such contexts, excluding partially corrupted samples can substantially diminish statistical power, especially in small or hard-to-replicate cohorts. This motivates the development of reconstruction strategies that aim to preserve samples while explicitly characterizing loci that exhibit unstable or distorted expression patterns.

The inability to obtain high-quality replicates can compromise the detection of genomic variations and may reduce the generalizability of analytical models, creating a pressing need for methodologies that can maximize the utility of existing data without resorting to exclusion of compromised samples. In this context, the proposed method offers a promising framework for identifying genomic regions affected by corrupted data while retaining valuable samples, thus addressing both the quantitative limitations of small datasets and the qualitative challenges posed by sample degradation. By preserving rather than discarding borderline or imperfect observations and systematically characterizing problematic regions, this approach may enhance statistical robustness and biological interpretability in studies where additional data acquisition is difficult, expensive, or unfeasible.

## 2 Materials and methods

### 2.1 Allele expression input data

Let $P=\left\{p_{1},p_{2},\ldots ,p_{m}\right\}$ be a population of m diploid individuals on which we run an RNA-seq experiment, involving a gene family $G=\left\{g_{1},g_{2},\ldots ,g_{n}\right\}$ : we assume each gene to be expressed at least once in P . For any g∈G , we denote by $A^{g}=\left\{A_{1}^{g},A_{2}^{g},\ldots ,A_{a\left(g\right)}^{g}\right\}$ the set of observed distinct alleles of g and by $E^{g}\in N^{a\left(g\right)\times m}$ the matrix depicting results of the experiment restricted to g , i.e., the read-counts of its alleles across P : column j of $E^{g}$ , associated to individual $p_{j}$ , has either one, two or zero non-null elements, depending on whether g is homozygously, heterozygously or not expressed in $p_{j}$ . Letting $\hat{a}=\sum_{g=1}^{n}a\left(g\right)$ the number of all observed distinct alleles, matrix $E\in N^{\hat{a}\times m}$ , obtained by vertically concatenating all such $E^{g}$ matrices, fully depicts the results of the experiment: for a column j , its first a(1) rows represent $g_{1}$ -alleles expression levels in $p_{j}$ , the following a(2) rows represent $g_{2}$ -alleles expression levels in $p_{j}$ and so on, as described in (Pagliarini et al., 2024). We define the *signature* of an individual p w.r.t. gene family G ^2^ as the (multi)set of all observed alleles in p (a multiset is required in order to explicitly depict the case of homozygously expressed genes. If g is one such gene for individual $p_{j}$ , then the unique expressed allele $A_{k}^{g}$ will have multiplicity 2 in $p_{j}$ ’s signature); furthermore, let the *genoset* of population P be the set of all signatures of its members. Individual $\hat{p}$ is *corrupted* if an error occurred during the RNA-seq experiment, so that at least one of $\hat{p}$ ’s expressed alleles and its relative read-counts could not be determined: thus, the expression vector $e_{\hat{p}}\in N^{\hat{a}\times 1}$ representing $\hat{p}$ will erroneously be 0 at an unknown number of positions.

### 2.2 Self-organizing maps

A SOM is a neural network model designed to project multidimensional data into a low-dimensional representation while preserving the topological structure of the input space. Unlike supervised networks, the SOM operates without explicit target labels, relying instead on competitive learning mechanisms and neighborhood-based adaptation rules. It is a nonlinear smooth mapping of high-dimensional input data onto the elements of a regular, low-dimensional array. Because of its ability to convert the nonlinear statistical relationships between high-dimensional data into simple geometric relationship of their image points on a regular two-dimensional grid of nodes, a SOM can be used for classification and visualization of high-dimensional data. This makes it particularly suitable for clustering, dimensionality reduction, and visualization of complex data distributions (Kohonen, 2013). In more details, SOMs represent a distribution of training data points using a finite set of models, which are automatically associated to nodes of a regular grid, such that more similar models (according to some metric) are closer in the grid than less similar ones, thus preserving the topological structure of the data. An input data point is then assigned to the node (cluster) whose associated model is most similar to it. The network architecture must be defined in advance: for most applications, arranging the nodes as a two-dimensional regular grid suffices to capture similarity relations of high-dimensional data; the optimal number of nodes may vary, depending on the coarseness of the relations one is interested in, but usually ranges between a dozen and a few hundred (Kohonen et al., 2001). A batch training procedure processes the entire training set iteratively. First, models $M_{i}$ , i.e., vectors in the same space of input data, are associated to each node i : while $M_{i}$ ’s can be randomly initialized, sampling them uniformly from the subspace spanned by the eigenvectors corresponding to the two largest principal components of the training set-so called *linear* initialization-yields faster convergence (Kohonen, 2013). Then, each training point is assigned to its most similar node. Each $M_{i}$ is iteratively recomputed as the mean of all training points assigned to either $M_{i}$ itself or to its neighbouring nodes, i.e., nodes within a certain distance from it: all models are updated concurrently, either for a fixed number of iterations or until cluster stability is reached, in a similar fashion to the classical k -means clustering algorithm (Kanungo et al., 2002).

### 2.3 Self-organizing maps implementation

We employ the SOM modelling and training tool included in the MATLAB®2024b release (Sobie, 2011), where the models are updated as previously described and the training stops after 200 batch-update epochs. Each SOM is implemented through a dedicated function, which automatically constructs the network architecture and initializes its parameters according to a set of defaults optimized for general-purpose use. In our experiment, the competitive layer follows a hexagonal topology, where each neuron is connected to six neighbors. This structure provides smoother neighborhood relationships compared to the square lattice, ensuring a more faithful preservation of local similarities in the mapping. The distance between neurons in the lattice is calculated using the link distance metric, which measures separation based on the number of edges connecting two units, rather than on their direct Euclidean distance. This choice supports stable neighborhood interactions during training.

The training process is based on the batch update algorithm described in subsubsection 2.3.1, which accumulates the influence of all input samples over an epoch and applies weight updates collectively at its end. This leads to improved convergence stability, reduced sensitivity to input ordering, and consistent results across repeated runs. The algorithm is divided into two phases.

An important characteristic of MATLAB’s SOM implementation is the absence of an explicit learning rate parameter. In many SOM formulations, the learning rate governs the magnitude of weight adjustments during training. In MATLAB®2024b, the learning dynamics are implicitly controlled by the neighborhood function and the number of epochs. This design simplifies model specification while preserving flexibility through the choice of map dimensions and training duration.

#### 2.3.1 Algorithm description

The goal of learning in SOM is to cause different parts of the network to respond similarly to certain input patterns. This is partly motivated by how visual, auditory or other sensory information is handled in separate parts of the cerebral cortex in the human brain. Unlike many other types of neural networks, a SOM does not need a target output to be specified. Instead, where the node weights match the input vector, that area of the lattice is selectively optimized to more closely resemble the data for the class the input vector is a member of. From an initial distribution of random weights, and over several iteration, the SOM eventually settles into a map of stable zones. Each zone is effectively a feature classifier. Therefore, the standard Kohonen learning algorithm is an unsupervised training process. It produces a vector quantizer by repeatedly updating the prototypes of the class-units, as described below. Let $x\left(t\right)\in R^{d}$ be an input vector at iteration t , and $w_{i}\left(t\right)\in R^{d}$ the weight vector associated with neuron i . The weights of the neurons are initialized either by randomly drawing in the input data space or sampled evenly from the subspace spanned by the two largest principal component eigenvectors. For each input vector, the best-matching unit (BMU) is identified as the neuron whose weight vector minimizes the Euclidean distance to the input (ordering phase):

$$i^{*}=\arg\underset{i}{\min}\left\|x\left(t\right)-w_{i}\left(t\right)\right\|.$$ (1)

Then, the weight vectors of the BMU and its neighboring neurons are updated according to (tuning phase):

$$w_{i}\left(t+1\right)=w_{i}\left(t\right)+\alpha \left(t\right)\;h_{i,i^{*}}\left(t\right)\;\left[x\left(t\right)-w_{i}\left(t\right)\right]$$ (2)

where α(t) is the learning rate, monotonically decreasing over time, and $h_{i,i^{*}}\left(t\right)$ is the neighborhood function, typically defined as a Gaussian:

$$h_{i,i^{*}}\left(t\right)=\exp\left(-\frac{{\left\|r_{i}-r_{i^{*}}\right\|}^{2}}{2\sigma^{2}\left(t\right)}\right).$$ (3)

Here, $r_{i}$ and $r_{i^{*}}$ denote the positions of neurons i and the BMU on the map lattice, while σ(t) represents the neighborhood radius, which decreases over time. The training procedure iterates over the dataset for a predefined number of epochs or until convergence. During training, both the learning rate α(t) and the neighborhood radius σ(t) decrease progressively, allowing the algorithm to transition from global ordering to local fine-tuning. After convergence, the SOM provides a topology-preserving mapping of the input space, enabling clustering through neuron activation patterns and a low-dimensional representation suitable for visualization and exploratory analysis.

### 2.4 Reconstruction problem and procedure

**FIGURE 1:**
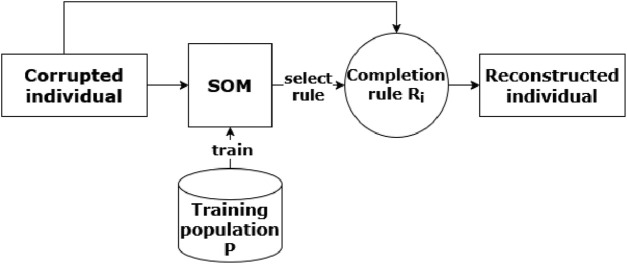
Overview of the proposed procedure, showing the reconstruction process of a corrupted individual $\hat{p}$ by using a SOM which associates $\hat{p}$ to a completion rule $R_{i}$ .

Our goal is to correctly reconstruct an individual p starting from its corrupted version $\hat{p}$ , i.e., we aim to identify p ’s missing alleles and their expression levels. We follow a two-phase strategy: first, a SOM, previously trained on an expression matrix $E\in N^{\hat{a}\times m}$ representing a population P of m -many uncorrupted individuals, assigns $\hat{p}$ to a cluster $C_{i}$ ; second, p is reconstructed applying a completion rule $R_{i}$ based on $P_{i}$ , the fraction of P the SOM assigns to the specific cluster $C_{i}$ . Figure 1 depicts the method.

**FIGURE 2:**
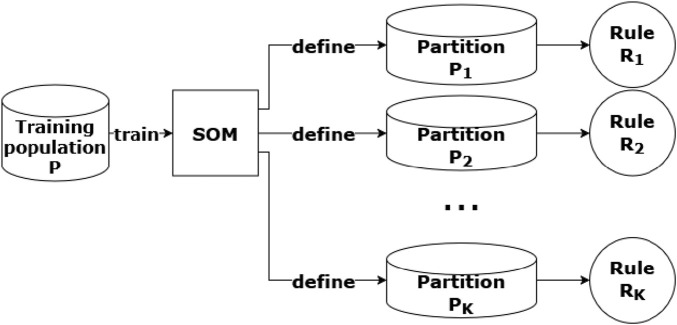
In a K -nodes SOM, K -many completion rules $R_{1},R_{2},\ldots ,R_{K}$ are defined, using the partitions of the training population P produced by the SOM.

Given a K -neuron SOM, K -many completion rules $R_{1},R_{2},\ldots ,R_{K}$ can be defined, as depicted in Figure 2.

According to this general definition, several reconstruction techniques could be applied for the second phase: we define a completion rule based on allele (-pair) frequency. Let g∈G be a gene expressed at least once in $P_{i}$ in which we assume that at least one error has occurred, i.e., we are uncertain about at least one of the g -alleles expressed in $\hat{p}$ : we denote by $\left({\hat{\alpha }}_{1},{\hat{\alpha }}_{2}\right)$ the pair^3^ of g -alleles in $\hat{p}$ and by $\left({\hat{r}}_{1},{\hat{r}}_{2}\right)$ their corresponding read-counts. Rule $R_{i}$ determines missing alleles and their expression levels according to which of the following three cases occurs.

If both ${\hat{\alpha }}_{1}$ and ${\hat{\alpha }}_{2}$ are unknown, we take $\left({\hat{\alpha }}_{1},{\hat{\alpha }}_{2}\right)=\left(\alpha_{1},\alpha_{2}\right)$ , the most frequent pair of g -alleles occurring in $P_{i}$ , and

$$\left({\hat{r}}_{1},{\hat{r}}_{2}\right)=\left(\left\lceil r_{\alpha_{1}|\alpha_{2}}\right\rceil ,\left\lceil r_{\alpha_{2}|\alpha_{1}}\right\rceil \right)$$ (4)

where $r_{\alpha_{j}|\alpha_{k}}$ is the average expression level of allele $\alpha_{j}$ when paired with $\alpha_{k}$ in $P_{i}$ .

If ${\hat{\alpha }}_{1}$ is known, two distinct subcases arise. If ${\hat{\alpha }}_{1}$ is observed at least once in $P_{i}$ , we compute a probability distribution π over all pairs of g -alleles of the form $\left({\hat{\alpha }}_{1},\alpha \right)$ occurring in $P_{i}$ :

$$\pi \left({\hat{\alpha }}_{1},\alpha \right)=\frac{\left|P_{i}\left({\hat{\alpha }}_{1},\alpha \right)\right|}{\left|P_{i}\right|}$$ (5)

where $P_{i}\left({\hat{\alpha }}_{1},\alpha \right)$ is the fraction of $P_{i}$ expressing $\left({\hat{\alpha }}_{1},\alpha \right)$ . We take the g -allele most commonly paired with ${\hat{\alpha }}_{1}$ in $P_{i}$ , that is

$${\hat{\alpha }}_{2}=argmax_{\alpha }\pi \left({\hat{\alpha }}_{1},\alpha \right)$$ (6)

and we assign the read-counts by computing:

$${\hat{r}}_{2}=\left\lceil {\hat{r}}_{1}\frac{r_{{\hat{\alpha }}_{2}|{\hat{\alpha }}_{1}}}{r_{{\hat{\alpha }}_{1}|{\hat{\alpha }}_{2}}}\right\rceil .$$ (7)

If ${\hat{\alpha }}_{1}$ never appears in $P_{i}$ , we compute the relative frequency f of each g -allele α that is observed at least once in $P_{i}$ . We take

$${\hat{\alpha }}_{2}=argmax_{\alpha }f\left(\alpha \right)$$ (8)

the most common g -allele in $P_{i}$ , and ${\hat{r}}_{2}=\left\lceil {\hat{r}}_{1}{\bar{r}}_{2}\right\rceil$ , with ${\bar{r}}_{2}$ being the average of all $\frac{r_{{\hat{\alpha }}_{2}|\alpha_{k}}}{r_{\alpha_{k}|{\hat{\alpha }}_{2}}}$ ratios such that pair $\left(\alpha_{k},{\hat{\alpha }}_{2}\right)$ appears in $P_{i}$ . We compute π over all pairs $\left({\hat{\alpha }}_{1},\alpha \right)$ , even those which do not appear in $P_{i}$ .

### 2.5 Validation indices

In order to evaluate our method, we consider test populations and their corrupted versions, in which each individual is modified by deleting the read-counts of a certain number of expressed alleles.

To assess SOM performance during the first phase, we analyze the behavior of two main metrics: *quality*, estimating how well the SOM is able to separate individuals into a clustering C which mirrors a given (ground-truth) labeling G , and *similarity*, estimating how well the SOM assigns individuals p and their corrupted versions $\hat{p}$ to the same cluster. To measure quality on a test population P , we employ 3 different indices, all ranging between 0 and 1, an higher value indicating a better clustering:using multiple indices is useful both to account for possible intrinsic biases and to highlight edge-cases in the dataset.

Simple Quality Index (SQI) is a metric we devised, which takes into account the largest intersections between each SOM cluster and all ground-truth classes (and viceversa). SQI is defined as:

$$SQI\left(P\right)=\sqrt{\underset{c\in C}{\sum }\frac{\max\left\{|c\cap g|:g\in G\right\}}{|c|\;numClusters}\cdot \underset{g\in G}{\sum }\frac{\max\left\{|c\cap g|:c\in C\right\}}{|g|\;numLabels}}$$ (9)

where c ’s are sets of individuals assigned to a given C -cluster, g ’s are sets of individuals of a given G -label. Each term in the first sum is the probability that the label of a random element of a cluster c is c ’s most representative label; similarly, each term in the second sum is the probability that a random individual of label g is assigned to g ’s most representative cluster.

Fowlkes-Mallows Index (FMI) is an external evaluation method used to determine the similarity between two classifications of the same dataset (Meila, 2007). FMI is defined as:

$$FMI\left(P\right)=\sqrt{\frac{TP}{TP+FP}\cdot \frac{TP}{TP+FN}}$$ (10)

where TP is the number of same signature-same label pairs of individuals, FP is the number of different label-same cluster pairs, FN is the number of same label-different cluster pairs. The first and second term of the radicand are the classic performance metrics known as *precision* and *recall*, respectively: FMI provides a balanced combination of the two.

Normalized Mutual Information (NMI) (Meila, 2007) is a measure of the mutual dependence between two random variables (X,Y) : intuitively, it measures how much knowing one variable reduces uncertainty about the other. Various normalized variants have been defined, taking into account the entropy of X and Y ; we consider the following:

$$NMI\left(P\right)=\frac{MI\left(G,C\right)}{\sqrt{H\left(G\right)H\left(C\right)}}$$ (11)

where MI(G,C) is the mutual information, H(C) the entropy of C , H(G) the entropy of G .

To assess similarity between P and its corrupted version $\hat{P}$ , we use Rand Index (RI) (Meila, 2007), which is defined as:

RIP,P^=TP+TNm2 (12)

where TP is the number of $\left(p,\hat{p}\right)$ pairs assigned to the same SOM cluster, TN the number of such $\left(p,\hat{p}\right)$ pairs assigned to different SOM clusters. Rand Index also ranges between 0 and 1.

To evaluate the full reconstruction procedure of an individual p , given $\hat{p}$ , we define two measures. First, letting $del\left(p,\hat{p}\right)$ the set of alleles expressed in p which are deleted in $\hat{p}$ and $guessed\left(p,\hat{p}\right)\subseteq del\left(p,\hat{p}\right)$ its subset which is correctly guessed by the completion rule, we compute *allele accuracy* $allAcc\left(p,\hat{p}\right)$ , ranging in the interval [0,1]:

$$allAcc\left(p,\hat{p}\right)=\frac{\left|guessed\left(p,\hat{p}\right)\right|}{\left|del\left(p,\hat{p}\right)\right|}.$$ (13)

Then we compute *read error* $readErr\left(p,\hat{p}\right)$ , a measure of how far predicted expression levels on correctly guessed alleles are from the real ones:

$$readErr\left(p,\hat{p}\right)=\frac{\sum_{\alpha \in guessed\left(p,\hat{p}\right)}\frac{\left|r_{\alpha }-{\hat{r}}_{\alpha }\right|}{r_{\alpha }}}{\left|guessed\left(p,\hat{p}\right)\right|}.$$ (14)

### 2.6 Assessing the influence of specific alleles

Starting from the concept of “feature-wise contribution to distance” introduced in (Kohonen, 1990), and considering the idea of “local exploratory analysis” (Ultsch and Siemon, 1990), we developed two *local feature relevance indices* in order to assess the influence of specific alleles on the topological placement of corrupted individuals. We computed these indices for each individual $\hat{p}$ after the training phase is complete. Let $BMU_{\hat{p}}$ and $sBMU_{\hat{p}}$ denote, respectively, the weight vectors of the *best* and *second Best Matching Unit* for a corrupted individual $\hat{p}$ . The first relevance metric, the *Local Error Contribution Index* $R_{\hat{p}}$ , quantifies the proportion of the distance between $\hat{p}$ and $BMU_{\hat{p}}$ contributed by each allele, i.e., how much it makes $\hat{p}$ diverge from the centroid of its assigned cluster. For a population with $\hat{a}$ observed alleles:

$$R_{\hat{p}}\left[k\right]=\frac{{\left(\hat{p}\left[k\right]-BMU_{\hat{p}}\left[k\right]\right)}^{2}}{\sum_{l=1}^{\hat{a}}{\left(\hat{p}\left[l\right]-BMU_{\hat{p}}\left[l\right]\right)}^{2}}\in \left[0,1\right]$$ (15)

where $\hat{p}\left[k\right]$ and $BMU_{\hat{p}}\left[k\right]$ are the k -th components of the individual and BMU weight vectors, respectively. A high $R_{\hat{p}}$ value identifies alleles in $\hat{p}$ that deviate significantly from the cluster expectation, possibly highlighting loci responsible for anomalies.

The second relevance metric, the *Discriminative Index* $D_{\hat{p}}$ , evaluates the role of each allele in the specific classification decision (i.e., assigning $\hat{p}$ to its BMU rather than its sBMU). We define a discriminative margin

$$\delta_{\hat{p}}\left[k\right]={\left(\hat{p}\left[k\right]-sBMU_{\hat{p}}\left[k\right]\right)}^{2}-{\left(\hat{p}\left[k\right]-BMU_{\hat{p}}\left[k\right]\right)}^{2}$$ (16)

and compute the normalized index as:

$$D_{\hat{p}}\left[k\right]=\frac{\delta_{\hat{p}}\left[k\right]}{\sum_{l=1}^{\hat{a}}|\delta_{\hat{p}}\left[k\right]|}\in \left[-1,1\right].$$ (17)

A positive value $\left(D_{\hat{p}}\left[k\right]>0\right)$ indicates that allele k actively supports the assignment to the winner $BMU_{\hat{p}}$ , while a negative value implies a contradiction favoring the runner-up $sBMU_{\hat{p}}$ . Together, these indices allow us to distinguish between features that drive quantization error and those that determine the decision boundary between competitive clusters: by computing their average across all considered individuals, we can estimate which alleles tend to be more relevant at a population level. In fact, as shown in (Pölzlbauer et al., 2006), regions of strong and coherent weight variation on a SOM correspond to meaningful structural boundaries in the data space. Therefore, features exhibiting pronounced and topologically consistent variations across the map can be considered discriminative.

### 2.7 Synthetic data generator

Given the limited size of several datasets, we develop a computational framework to generate synthetic individuals from the input data of allele expression introduced in Subsection 2.1. Our generator is based on the integration of mating process of organism with genetic algorithms. The method takes as input a dataset containing, for each individual and variant, an index identifying the observed allele and the corresponding allele-specific read count. Formally, this input is represented by the matrix $E\in N^{\hat{a}\times m}$ and its Boolean form $E^{b}$ , with $E_{ij}^{b}=1$ if and only if $E_{ij}\neq 0$ . The generator loads the chromosome-specific dataset and extracts the allele index and read count matrices. Synthetic individuals are generated through a two-stage process. In the first stage, the original population is iteratively expanded using an evolutionary meta-heuristic that combines parental recombination, mutation, and fitness-based selection. In the second stage, a Sequential Monte Carlo (SMC)–based procedure is applied to further augment the dataset while preserving empirical allele-specific count distributions.

#### 2.7.1 Evolutionary generation of synthetic individuals

The mating selection process is carried out randomly following the procedure developed in (Shuster, 2009), that is based on the probability of sexual selection. At each iteration of the evolutionary procedure, two parents are selected from the current population. Each parent is represented as an $\hat{a}\times 2$ matrix, where rows correspond to alleles across loci, the first column encodes allele indices, and the second column encodes allele-specific read counts. To modulate the recombination process, a scalar mating index is computed as:

$$I_{\text{mates}}=1+\left(\sigma_{p_{m}^{T}}^{2}-\sigma_{p_{f}^{T}}^{2}\right)$$ (18)

where $\sigma_{p_{m}^{T}}^{2}$ and $\sigma_{p_{f}^{T}}^{2}$ denote the variance of the selected parents to be mated at iteration T . This index provides a coarse measure of relative allelic variability and determines which parent is treated as the primary contributor during recombination. This expression is essential in order to produce new offspring at the iteration T where $I_{\text{mates}}$ can be positive or negative. To produce new offspring $p_{c}^{T}$ , the Hardy-Weinberg (HW) principle is employed (Edwards and Hardy, 1908; Hardy, 1908; Weinberg, 1908), with:

$$p_{c}^{T}=\left\{\begin{array}{cc} \alpha ⊙p_{m}^{T}+\beta ⊙p_{f}^{T} & \text{if }I_{\text{mates}}\geq 0\\ \beta ⊙p_{m}^{T}+\alpha ⊙p_{f}^{T} & \text{if }I_{\text{mates}}<0 \end{array}\right.$$ (19)

where α is a normal random distribution, β=1−α and ⊙ is the element-wise product. The expressions of α and β refer to the concept of HW principle, where the sum of α⨁β=1 . α and β can be assumed as allele inherited from father and mother, respectively. Therefore, offspring generation is performed under an HW random mating assumption, where for each allele position, a uniform random is drawn and converted into two complementary Bernoulli indicators ensuring that exactly one of the two indicators is active. When $I_{\text{mates}}\geq 0$ , the offspring inherits, at position ℓ , the allele index and corresponding read count from the father if α(ℓ)=1 , and from the mother if β(ℓ)=1 . When $I_{\text{mates}}<0$ , parental roles are swapped and the same Bernoulli scheme is applied with the mother treated as the primary contributor. As a result, each locus independently inherits allelic information from one of the two parents with probability 0.5, producing an offspring whose allele-specific profile is a mosaic of the parental profiles while preserving Mendelian-like segregation. The resulting offspring is subsequently perturbed by mutation. A fixed mutation rate of 3% of the loci is used, and three mutation operators are available: *i)* a *scramble* mutation, which randomly permutes a subset of alleles along the chromosome; *ii)* a *swap* mutation, which exchanges the alleles at two randomly chosen positions; and *iii)* a *random resetting* operator, which replaces selected alleles with alternative states informed by the empirical read count distribution (Goldberg, 1989; Mitchell, 1996). For each iteration, multiple mutated offspring candidates are generated. To ensure biological plausibility, each candidate offspring is evaluated using a fitness function based on the deviation of its allele-specific read count vector from the empirical population counts. The offspring minimizing the Euclidean distance is selected and appended to both the allele index and read count matrices, thereby expanding the population by one synthetic individual per iteration. Throughout this process, the number of allelic states observed at each locus in the original dataset is tracked to constrain allele assignments to feasible values. After completing the evolutionary expansion, the extended allele index and read count matrices are mapped into a compact individual-by-variant representation. Newly generated individuals are assigned unique synthetic labels and concatenated with the original sample identifiers.

#### 2.7.2 Sequential monte carlo-based augmentation

To further increase sample size while preserving the empirical distribution of ASE counts, a SMC-based synthetic data generator is applied to a randomly selected subset of the individuals. Approximately half of the individuals are selected at random, and their allele-specific read count profiles are used as input. For each feature (i.e., each allele-specific count across loci), the observed counts are treated as a univariate empirical distribution. A large set of particles is initialized by resampling from the original data, and synthetic samples are generated iteratively through a resampling and reweighting scheme. At each step, particle weights are updated as a decreasing function of the distance between the particles and the newly generated synthetic value, promoting consistency with the empirical distribution while avoiding over-concentration on high-frequency observations. The resulting synthetic count matrix is converted into a corresponding allele matrix via a sign transform, encoding the presence or absence of allele-specific signal. Typically, an additional number of synthetic individuals corresponding to approximately 30% of the size of the selected subset is generated and appended to the existing dataset, with new synthetic labels assigned accordingly. The final output consists of a combined dataset including the original individuals, the evolutionary meta-heuristic–derived synthetic individuals, and the SMC-derived synthetic individuals, together with their allele index matrix, read count matrix, and updated individual labels. This framework enables controlled augmentation of ASE datasets while preserving key distributional properties of allele-specific expression across loci.

## 3 Results

To assess the performance of both phases of our SOM-based procedure, we test it in different settings, measuring the aforementioned evaluation indices as the number of deletion errors increases.

**FIGURE 3:**
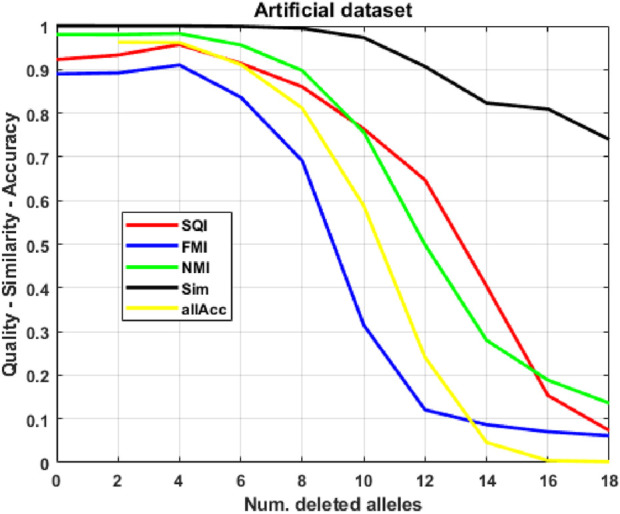
Quality, similarity and accuracy. Clustering quality, clustering similarity and allele prediction accuracy on corrupted populations, as the number of deleted alleles increases.

First (Figure 3), we assess performance on artificially generated data, simulating RNA-seq experiments. In this setting, we have many individuals generated from a limited number of signatures, which are used as the ground-truth labeling G .

**FIGURE 4:**
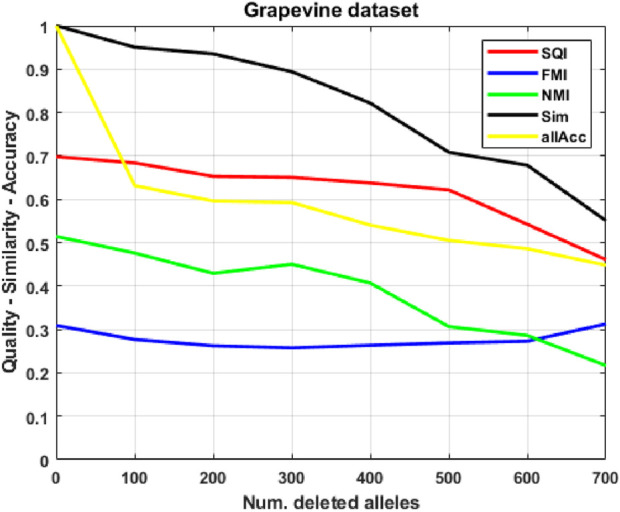
Clustering quality and similarity on corrupted versions of the grapevine population, as the number of deleted alleles increases.

Next, following on the promising results of the first test, we apply our SOM-based procedure to a real dataset derived from 98 cultivars representative of the variability present in *Vitis vinifera*, by employing read counts of genes in chromosome 1 of leaves. In this case, since each individual has a unique signature, we require a different ground-truth labeling G to evaluate the SOM clustering: we use the labels produced by a 2-phase clustering procedure (henceforth called 2 PP), based on spectral clustering, which we developed in a previous work (Pagliarini et al., 2026). In such a work, we showed that such labeling has biological significance, justifying its use as a ground-truth, given the lack of alternatives. In this much more complex instance (Figure 4), while the performance of our quality indices is significantly worse than in the artificial case and further degrades as the amount of corruption increases, the method still displays remarkable allele accuracy, correctly guessing more than half the deleted alleles for up to 500 deletions (more than 60% for up to 300 deletions).

**FIGURE 5:**
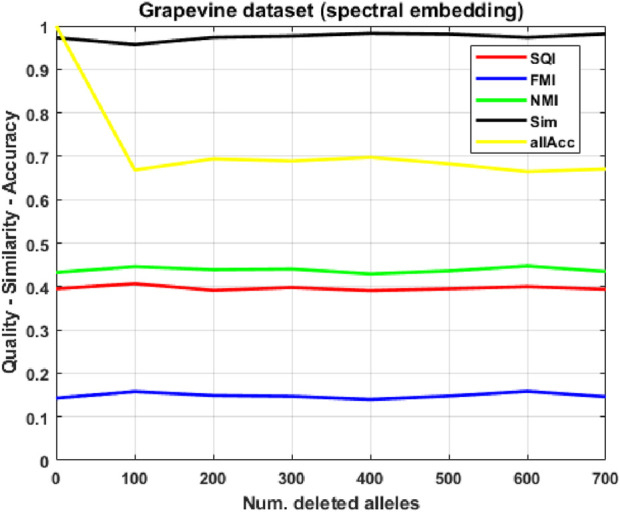
Clustering quality and similarity on corrupted versions of the grapevine population, using the *spectral embedding*, as the number of deleted alleles increases.

Attempting to improve our results, instead of training the SOM directly on high-dimensional sparse vectors of allele counts, we compute an embedding of the population in a low-dimensional spectral space (the same used by 2 PP), where individuals are mapped based on their global connectivity patterns within a distance graph (the distance being a function of the amount of common expressed alleles in their signatures). Using such spectral embedding (Figure 5), we observe an improvement in allele accuracy (∼70%) .

**FIGURE 6:**
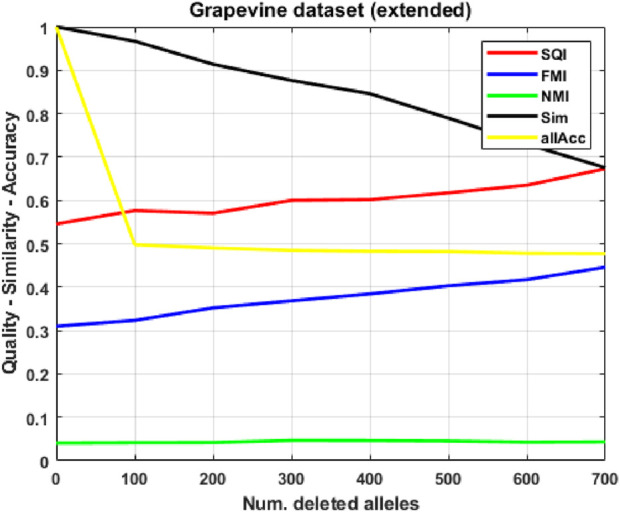
Clustering quality and similarity on corrupted versions of the extended (real plus synthetic) grapevine population, as the number of deleted alleles increases.

**FIGURE 7:**
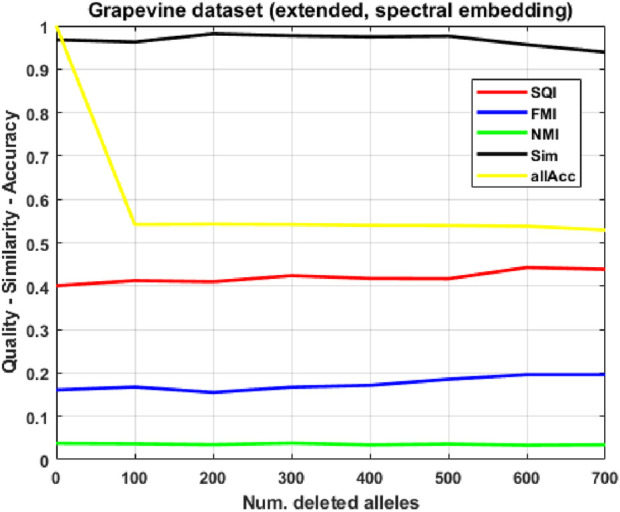
Clustering quality, similarity and allele accuracy on corrupted versions of the extended (real plus synthetic) grapevine population, using the spectral embedding, as the number of deleted alleles increases.

Assuming that the limited size of the population compared to the feature space (more than 16.000 features per individual, most of which are equal to 0) might hinder the SOM training, we extend P by adding synthetic individuals created by the generator described in Subsection 2.7. Here, two of the quality indices display unexpected behavior (Figure 6), which we explain to be due to biases in both the extended dataset and the two indices, while allele accuracy worsens (<50%) . Applying the spectral embedding method to the extended dataset (Figure 7), we observe a slight improvement in allele accuracy, confirming this approach to be the most appropriate for our task. Finally, on the read-count representation of the real-only dataset, we compute Z-scores, derived from the two relevance indices, to determine which alleles contribute in a statistically significant way to the displacement of individuals in the SOM.

### 3.1 Artificial dataset

We suppose an RNA-seq experiment on m individuals to involve n=10 genes, such that 10 different alleles are observed for each gene (thus having expression matrix $E\in N^{\mathrm{100\times m}}$ ). We create *training population* P of size m=5000 individuals by randomly generating a genoset of 100 distinct signatures, which will also be used as the ground-truth labeling G : each signature contains 20 alleles, 2 for each gene. For each individual, we randomly select its signature from the training genoset and assign a read-count value, randomly chosen from range [1,700], to each of its alleles, so that the resulting P will display an almost uniformly distributed representation of the whole genoset. To evaluate the net, we create test populations and their corrupted versions, in which each individual is modified by deleting the read-counts of a certain number of expressed alleles. Test populations of 1,000 individuals are generated as previously described, sharing the same genoset as the training population. For the SOM topology, we choose a 10x10 grid, so that the network has K=100 neurons, as many as the signatures in the genoset. To account for fluctuations in results due to randomness, 15 networks are trained on distinct training sets and each SOM is tested on 100 test populations.

We consider corrupted populations and study the evolution of quality and similarity indices as a function of numDel , the number of deleted alleles per individual, ranging from 0 to 18; furthermore, we also assess allele accuracy allAcc during the reconstruction phase. Quality, similarity and accuracy indices are averaged across all 100 test populations of each SOM: such means are then further averaged across all 15 networks. Figure 3 shows how indices remain fundamentally stable even for a considerable number of deleted alleles (6-8, equivalent to 30%–40% of the whole signature): such results suggest that SOMs are quite robust to errors, since they mostly assign individuals and their corrupted versions to the same cluster. Furthermore, the procedure correctly guesses most alleles. Since read counts are generated randomly, index readErr is not meaningful in this context and is therefore ignored for this preliminary assessment.

### 3.2 Grapevine variants dataset

Following the promising results of the first test, we applied our SOM-based procedure to a previously developed grapevine variant database (Magris et al., 2021). In this work, berries and leaves samples from 98 cultivars representative of the variability present in *Vitis vinifera* have been collected, used for library preparation, and then sequenced. Particularly, we employed genes belonging to chromosome 1 of leaves. It is important to point out that a set of 1/19 of genes represents a random sample even if it corresponds to a single chromosome: this is due to the fact that there are many more differences within a chromosome then between different chromosomes that can impact on allele imbalance, such as, for instance, chromatin state or gene expression levels. In this dataset, 1,566 genes are considered, with 10.92 possible alleles per gene, on average; furthermore, only 106 genes have less than 4 possible alleles. Such amount of variability confirms the non-trivialness of the allele prediction task. As stated previously, we use the labels produced by 2 PP as ground-truth. In short, the 2 PP procedure relies on spectral clustering. In its first phase, it constructs a distance graph of the population, where distance between two individuals is defined according to the number of common expressed alleles between the two (*Jaccard index*); then, it computes the *Normalized Symmetric Laplacian* of such graph and performs eigen-decomposition to extract the top K eigenvectors (where K is the number of estimated clusters); these eigenvectors define a low-dimensional spectral space, where individuals are mapped based on their global connectivity patterns; finally, k -means clusters the population in the spectral space. The same process is applied a second time on each resulting subpopulation, using a different distance metric, in order to further refine them.

One difficulty with the standard Kohonen algorithm is the need of *a priori* knowledge of the number of classes to be separated during the classification process. On this specific grapevine dataset, 2 PP partitions P into 15 classes (which are shown to be biologically significant, justifying using this labeling as a ground-truth G ): thus, we set a 4×4 SOM, in order to have an almost equal number of neurons, while maintaining a grid structure.

To limit the variability of the number of expressed alleles when corrupting our population, we apply a filtering, keeping only those (82) individuals which express at least 700 alleles: knowing that no p∈P expresses more than 1,500 alleles, this grants the resulting subpopulation $P_{\geq 700}$ to be balanced. After training the SOM on P , we study the evolution of our parameters, using $P_{\geq 700}$ as a test population, by deleting {0,100,…,700} alleles from each individual. Figure 4 depicts the results: the performance of our quality indices is significantly worse than in the artificial case and further degrades as the amount of corruption increases, but the method still displays remarkable allele accuracy, correctly guessing more than half the deleted alleles. Average read error, reported in Table 1, varies significantly, growing from 27% (100 deletions) to 158% (700 deletions). Overall, results show our reconstruction rules to be reasonably effective at guessing alleles and estimating read counts at relatively low corruption levels, with performance on the latter task degrading rapidly as deletions increase.

**TABLE 1:** Average read-count reconstruction error on the grapevine datasets.

Deletions	Real	Real (spectral)	Extended	Extended (spectral)
100	0.27	0.44	47.87	44.84
200	0.50	0.37	41.67	32.69
300	0.63	0.44	43.70	40.10
400	0.87	0.52	46.92	39.49
500	1.09	0.61	35.74	39.73
600	0.73	1.01	45.73	37.55
700	1.59	0.76	35.49	40.86

Index *readErr*, as defined in Subsection 2.5, measures the relative distance between predicted and actual read counts on correctly predicted alleles (e.g., a value of 1.2 means that, on average, predicted reads are equal to 2.2 the actual reads). Results are divided by number of deleted alleles on each individual, i.e. 0, 100, … , 700.

### 3.3 Grapevine variants dataset (spectral embedding)

The raw allele-count representation extends over a huge feature space (over 16,000 alleles) compared to the size of the dataset, with most features being equal to 0. In order to improve performance, we attempt to redefine individuals according to spectral coordinates, following the same process employed in the first phase of the 2 PP procedure, described in Subsection 3.2. Instead of training the SOM on high-dimensional sparse vectors of allele counts, we first compute a spectral embedding of each individual in P , defined by the top 16 eigenvectors of the Laplacian matrix of the distance graph, where 16 is again the number of neurons in the SOM to be trained. Then, to evaluate corrupted samples from $P_{\geq 700}$ without recalculating the entire graph spectrum, which would differ for every corruption level, we employ the *Nyström Extension* (Fowlkes et al., 2004), a technique which projects corrupted individuals directly into the existing eigenspace defined by the training data, ensuring that the SOM classifies data according to their adherence to the original biological structure. Looking at Figure 5, one can see that quality indices SQI and FMI perform much worse than in the raw-representation case, while NMI is only slightly lower for few (≤100) deletions, but remains stable as corruption increase. This stabilization phenomenon actually extends to all indices: similarity and allele accuracy remain constantly close to 1 and 0.7, respectively. Overall, while the spectral representation behaves worse with respect to matching the 2 PP classification, it seems to be much more robust to corruption, while also being more accurate when it comes to our main task, missing allele prediction; furthermore, the average read error (Table 1) varies from 36% to 101% , appearing to be more limited than in the read-count representation case, although still high.

### 3.4 Extended grapevine variants dataset

We apply our generator to obtain an extended population P consisting of 1,148 individuals, including the 95 real ones employed in the previous analysis. We repeat the exact same experiment performed for the original population, keeping only the 1,048 individuals with at least 700 expressed alleles as the test population $P_{\geq 700}$ . Figure 6 shows the results: compared to the real-only case, allele accuracy is worse (∼0.5) , but less sensitive to deletions, and similarity behaves almost identically. Quality indices require a finer analysis: SQI and FMI, counterintuitively, increase linearly with the number of deletions. This can be explained by a bias in both the indices and the synthetic population generator: the way they are defined, FMI and SQI are sensitive to class imbalance and fusion, meaning their values tend to be boosted when a single superclass of one labeling contains a vast majority of those individuals belonging to one or more classes of the compared labeling. In this case, the 2 PP-derived ground-truth G had 3 major classes encompassing 538, 213 and 143 individuals: this highlights a possible issue in our synthetic generator, which leads to the creation of many “average” individuals making up the majority of the extended population; on the uncorrupted population, the SOM clustering C consistently has one superclass of ∼600 individuals, with most individuals coming from those 3 major G -classes; when 700 deletions occur, the C -superclass grows to ∼850/900 individuals, with the additional ones again coming from the same G -classes, thus exacerbating this accumulation phenomenon and boosting SQI and FMI (since the number of true positives, i.e., pairs from the same G -class within the same C -class, is maximized). On the other hand, NMI correctly detects this imbalance, showing that the actual mutual information between G and C is almost null.

In general, extending the population did not yield the desired improvements, effectively worsening missing allele prediction, and highlighted biases in the generator and the SQI/FMI quality indices, showing NMI to be the most reliable one. Average read error (Table 1) varies between 3300% and 4800% , an exceedingly large amount: most likely, this is caused by an issue occurring in our synthetic generator which leads to outliers with disproportionately high read-counts; we plan to investigate this in the near future.

### 3.5 Extended grapevine dataset (spectral embedding)

Our previous analysis reveals that standard count-based SOM clustering of the extended population is susceptible to a homogenization artifact driven by severe class imbalance: the synthetic generation process introduced a dominant sub-population that overwhelmed the biological signal, causing the SOM to collapse most individuals into a single sink cluster, resulting in erroneously high quality metrics (SQI, FMI) despite NMI correctly indicating a loss of biological structure. Furthermore, this phenomenon was further exacerbated as the corruption level increased.

Out of completeness, we analyze what happens when the spectral embedding is used for the extended dataset. Figure 7 shows that the stabilization effect across corruption level, observed for the real-only dataset, persists. The biased behavior of quality indices SQI/FMI is mitigated, while NMI remains close to 0, confirming that the SOM clustering C is completely uncorrelated to the 2 PP labeling G on this extended dataset; we also point out that allele accuracy slightly improves compared to the raw representation case of the same dataset (by a margin of ∼5% ), but remains inferior to the results obtained by the original real-only dataset (with both representations). Read error remains in the same, unusually high range of the raw representation case.

### 3.6 Relevant genes analysis

For the real-only grapevine dataset, using the raw read-count representation, we also compute relevance indices $R_{\hat{p}}$ (local error contribution) and $D_{p}$ (discriminative index) for each corrupted individual $\hat{p}$ , as defined in Section 2. We recall that $R_{\hat{p}}\left[k\right]\in \left[0,1\right]$ quantifies the contribution of allele k to the divergence of $\hat{p}$ from the centroid of its assigned cluster, while $D_{\hat{p}}\left[k\right]\in \left[-1,1\right]$ quantifies how much allele k actively supports (positive value) or undermines (negative value) the assignment of $\hat{p}$ to its cluster centroid $BMU_{\hat{p}}$ . We compute allele-wise mean values of both indices for each corrupted test population $\hat{P}$ , i.e.,

$$R_{\hat{P}}\left[k\right]=\frac{\sum_{\hat{p}\in \hat{P}}R_{\hat{p}}\left[k\right]}{\left|P\right|},\;D_{\hat{P}}\left[k\right]=\frac{\sum_{\hat{p}\in \hat{P}}D_{\hat{p}}\left[k\right]}{\left|P\right|}.$$ (20)

Similarly to what we have done previously for other indices, such means are further averaged across all test populations with the same number of deletions, in order to account for fluctuations due to randomness in the selection of deleted alleles.

For a given corruption level, let us denote such final mean vectors by R and D , respectively. We convert R and D into *z-scores* Z[R,k] and Z[D,k] , which measure the number of standard deviations by which R[k] and D[k] are above or below mean(R) and mean(D) , respectively. We then follow a Z-test, selecting the critical value of 1.96 for a 95% confidence level (Montgomery and Runger, 2014). Since we are interested in values that are high and statistically significantly different from the mean, we filter all those alleles that are on the right side of the distribution of local error contribution (i.e., Z[R,k]≥1.96 ) for at least one corruption level and map them back to their genes employing the STRING Database v12.0 (Szklarczyk et al., 2023), identifying 22 genes that encompass 75 alleles. Details are reported in Table 2.

**TABLE 2:** Mapping between the 22 genes having a statistical significant local error contribution and STRING database entries.

Gene name	STRINGdb ID
VIT_01S0010G00990	F6HGG6
VIT_01S0010G02280	F6HG36
VIT_01S0010G03620	D7TAY3
VIT_01S0011G02710	A5AEV3
VIT_01S0011G02720	D7T9B8
VIT_01S0011G02860	F6HF24
VIT_01S0011G03010	F6HF12
VIT_01S0011G03200	D7T981
VIT_01S0011G03590	D7T943
VIT_01S0011G05370	F6HFF7
VIT_01S0011G06250	D7T8G6
VIT_01S0026G00570	D7TNN1
VIT_01S0026G01050	A5C631
VIT_01S0026G01460	D7TNF9
VIT_01S0026G02480	F6HPR1
VIT_01S0127G00060	D7TDL2
VIT_01S0127G00450	F6HHV6
VIT_01S0127G00470	F6HHV4
VIT_01S0127G00720	D7TDF1
VIT_01S0127G00740	F6HHU0
VIT_01S0137G00210	F6GVJ9
VIT_01S0150G00130	D7TFI8

**FIGURE 8:**
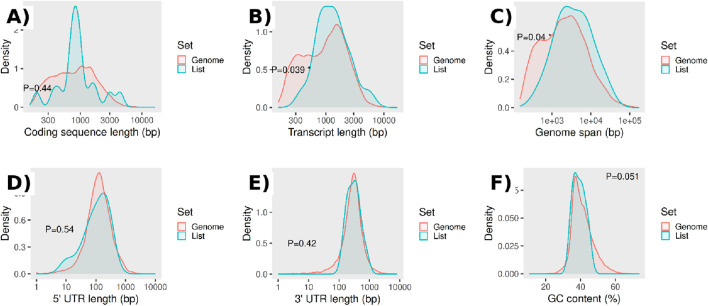
Gene feature distributions for the input gene list compared with the genomic background. Kernel density estimates for **(A)** coding sequence length (bp), **(B)** transcript length (bp), **(C)** genomic span (bp), **(D)** $5^{\prime }$ UTR length (bp), **(E)** $3^{\prime }$ UTR length (bp), and **(F)** GC content (%). The input gene list (*List*, cyan) is contrasted with the genome-wide reference set (*Genome*, red). P-values reported in each panel indicate the significance of differences between the two distributions (two-sample comparison). Overall, transcript length **(B)** and genomic span **(C)** show statistically significant shifts, whereas coding sequence length and UTR lengths are broadly comparable between sets **(A,D,E)**, with GC content showing a borderline difference **(F)**.

To assess whether these genes exhibit structural or compositional biases relative to the genomic background, we compared multiple gene features using kernel density estimates (Figure 8). Coding sequence length distributions were highly comparable between the two sets (Figure 8A), indicating no bias in the length of protein-coding regions. Similarly, no significant differences were observed for $5^{\prime }$ and $3^{\prime }$ untranslated region lengths (Figures 8D,E), suggesting that regulatory regions flanking the coding sequence are not systematically altered in the input gene list. In contrast, transcript length and genomic span showed modest but statistically significant differences (Figures 8B,C), with the input gene list displaying a shifted distribution relative to the genome-wide reference. These results indicate that genes in the input list tend to exhibit distinct transcriptional architectures, potentially reflecting functional or regulatory properties specific to the biological context under investigation. GC content distributions were largely overlapping between the two sets (Figure 8F), although a borderline shift was observed, suggesting a weak tendency toward altered nucleotide composition. Overall, these comparisons indicate that the input gene list is not affected by trivial annotation biases but instead displays specific structural characteristics, supporting the biological relevance of the selected genes.

**FIGURE 9:**
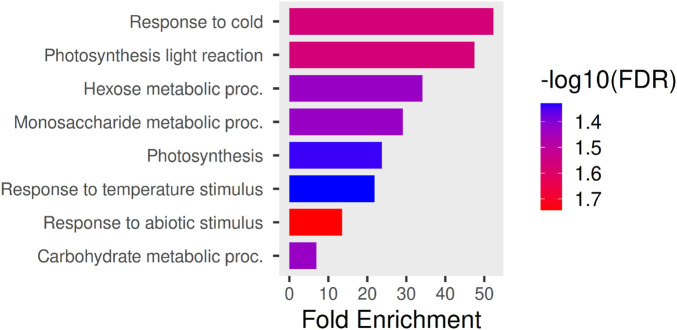
Gene Ontology (GO) enrichment analysis of the gene having a significant Local Error Contribution Index. The bar plot shows significantly enriched (FDR≤0.05) GO Biological Process terms for the input gene list. The x-axis indicates fold enrichment relative to the genomic background, while bar colors represent statistical significance expressed as $-\log_{10}\left(FDR\right)$ . Enriched terms are predominantly associated with responses to cold and temperature stimuli, photosynthesis and photosynthetic light reactions, and carbohydrate metabolic processes, indicating a coordinated functional signature linking environmental stress response and energy metabolism.

Functional enrichment analysis (Ge et al., 2019), based on the complete reference genome for grapevine developed in (Shi et al., 2023), of the 22 genes revealed a strong over-representation of biological processes related to environmental stress responses and energy metabolism (Figure 9). Notably, terms associated with cold and temperature responses, as well as broader abiotic stress responses, showed the highest fold enrichment and statistical significance. In addition, multiple photosynthesis-related processes, including the light reactions, were significantly enriched, together with carbohydrate and monosaccharide metabolic processes. These results suggest that the selected genes are functionally coordinated and participate in an integrated response linking environmental stress adaptation with metabolic and photosynthetic regulation. Together with the absence of major structural biases observed at the gene architecture level (Figure 8), these enrichment results support the biological relevance of the input gene list and argue against technical selection effects.

We do the same for the discriminative index, including statistically significant alleles on the left side (i.e., Z[R,k]≤−1.96 ), since we are also interested in alleles that tend to contribute *negatively* to the final SOM assignment: in this case, we identify 9 genes associated with positive valued alleles and 1 gene associated with a negative value. They are reported in Table 3.

**TABLE 3:** Mapping between the genes having a statistical significant discriminant index and STRING database entries.

Gene name	STRINGdb ID
VIT_01S0010G03620	D7TAY3
VIT_01S0011G02710	A5AEV3
VIT_01S0011G02860	F6HF24
VIT_01S0011G03010	F6HF12
VIT_01S0026G01050	A5C631
VIT_01S0026G02480	F6HPR1
VIT_01S0127G00470	F6HHV4
VIT_01S0127G00740	F6HHU0
VIT_01S0137G00210	F6GVJ9

**FIGURE 10:**
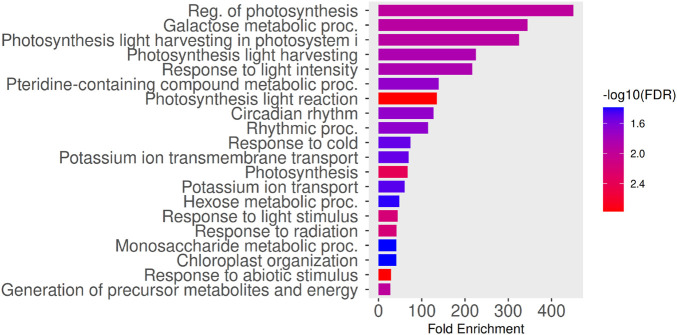
Gene Ontology (GO) enrichment analysis of genes having significant discriminative index. The bar plot shows significantly enriched (FDR≤0.05) GO Biological Process terms. The x-axis indicates fold enrichment relative to the genomic background, while bar colors represent statistical significance expressed as $-\log_{10}\left(FDR\right)$ . Enriched terms are predominantly associated with the regulation of photosynthesis, light harvesting and photosystem I activity, responses to light intensity and radiation, circadian rhythm, and potassium ion transport, indicating a specialized regulatory program linking photosynthetic control, environmental signal integration, and metabolic coordination.

In this case, enrichment analysis revealed a more specialized functional signature compared to the previous set of genes (Figure 10). The most significantly enriched terms were associated with the regulation of photosynthesis, including light harvesting processes and photosystem I activity, as well as responses to light intensity and radiation. Notably, circadian rhythm–related processes were also overrepresented, suggesting temporal regulation of photosynthetic and metabolic functions. In addition, enrichment of potassium ion transport and carbohydrate metabolic processes indicates tight coupling between photosynthetic regulation, ion homeostasis, and energy metabolism. While stress-related terms such as response to cold and abiotic stimulus remained detectable, they appeared in the context of a more refined regulatory program, highlighting functional specialization within gene sets derived from the same experimental framework.

### 3.7 Replacing SOMs with k -means

As a baseline reference, we repeated the same reconstruction experiments on the real grapevine dataset, using both the raw read-counts and spectral representations, replacing SOMs with the standard k -means algorithm (Jain et al., 1999) in the clustering phase, keeping k=16 for consistency. Results are fundamentally identical to those obtained with SOMs and observed in Figures 4, 5, thus we omit them for brevity.

Let us analyze the convergence between the two approaches when k=16 . Under this condition, both algorithms define a set of 16 prototype vectors: $\left\{w_{1},\ldots ,w_{16}\right\}$ . In k -means, these vectors correspond to centroids that minimize the within-cluster sum of squares, while in a SOM, each neuron is associated with a weight vector updated according to Equation 2. Thus, both methods partition the data space through nearest-prototype assignment. It is possible to see that the theoretical link between SOM and k -means lies in the neighborhood function Equation 3. As training progresses, σ(t)→0 , and in the limit:

$$h_{i,i^{*}}\left(t\right)=\left\{\begin{array}{cc} 1 & i^{*}=i\\ 0 & i^{*}\neq i. \end{array}\right.$$ (21)

Under these assumptions, the update rule simplifies to $w_{i}\left(t+1\right)=w_{i}\left(t\right)+\alpha \left(t\right)\left[x\left(t\right)-w_{i}\left(t\right)\right]$ , which corresponds to rule used in k -means. Therefore, as the neighborhood radius in a SOM shrinks to zero, or becomes sufficiently small, the update rule effectively only modifies the BMU. At this limit, the SOM algorithm reduces to a competitive learning process known as Forgy’s algorithm or basic competitive vector quantization, which minimizes the same quantization error (mean squared error) as k -means (Ritter, 1991). More on the comparison between SOMs and k-means, as well as on the advantages of using the former can be find in Section 4.

## 4 Discussion

In this work, we introduced a technique based on SOMs for the reconstruction of missing allele-specific expression data and the exploration of genomic anomalies.

It is important to clarify the methodological scope of the proposed framework. The anomaly detection component of our approach should not be interpreted as a copy-number or allelic-shift caller in the conventional sense. Unlike methods such as eSNP-Karyotyping, CaSpER, or RNAseqCNV, which explicitly model dosage alterations and perform genomic segmentation, our SOM-based indices quantify feature-wise contributions to topological displacement within an unsupervised embedding. Consequently, the regions identified through the local error contribution and discriminative index represent loci that induce geometric distortion in the learned representation of ASE profiles, rather than explicit estimates of chromosomal gains, losses, or allelic dosage imbalance. In this sense, our framework should be regarded as complementary to CNV-oriented pipelines. While segmentation-based tools focus on detecting structured genomic dosage alterations, our method emphasizes reconstruction robustness and internal consistency of ASE signatures in high-dimensional sparse settings, particularly in small-cohort contexts.

Our initial validation on artificially generated populations demonstrated the theoretical robustness of the approach, where the network successfully recovered over 90% of missing alleles even under significant data deletion. However, the application to the *Vitis vinifera* dataset revealed some limitations of standard distance-based clustering when applied to high-dimensional and sparse genomic data, with allele accuracy ranging between 50% and 60% . We remark that, in this ASE dataset, 1566 genes are considered, with 10.92 possible alleles per gene, on average; furthermore, only 106 genes have less than 4 possible alleles, making the missing allele identification task non-trivial.

We hypothesized that degradation of performance on the real-world dataset might be related to the sparsity of raw allele-count vectors, hindering the ability of the map to preserve topological stability as corruption increases. To address these challenges, we implemented a spectral embedding strategy that projects individuals into a low-dimensional space defined by the eigenvectors of a Laplacian matrix. This transformation shifted the learning task from raw local features to global connectivity patterns derived from a Jaccard-based distance graph. The use of this spectral representation significantly improved allele accuracy to approximately 70% and made the reconstruction metrics highly robust to data corruption.

Our investigation into dataset augmentation using a synthetic generator based on evolutionary and Sequential Monte Carlo processes provided critical insights regarding validation metrics and our generator. While the extended dataset did not improve reconstruction performance, it exposed a specific bias in the SQI and FMI quality indices. These metrics exhibited a counter-intuitive increase as corruption levels rose, driven by the creation of a huge sub-population that caused the SOM to collapse distinct classes of the reference ground-truth labeling G into a single superclass. On the other hand, the NMI index correctly identified the complete loss of correlation between ground-truth classes and SOM clusters, underscoring the need for further work on our generator.

Despite the improvements in allele accuracy achieved through spectral embedding, the estimation of specific expression levels remains prone to significant error, highlighting the need to reconsider this part of our reconstruction rules. The defined completion rule is based on allele (or allele-pair) frequency. Importantly, these rules are not heuristic but are grounded in well-established biological principles derived from population genetics, particularly the expected distribution of allele frequencies under evolutionary and demographic constraints. By leveraging these biologically informed assumptions, the completion strategy preserves plausible genotype configurations while minimizing the introduction of artificial bias. Moreover, we would like to point out that our rule operates as a cluster-conditional smoothing procedure and does not attempt to model the full dispersion structure of RNA-seq read counts, which would require a probabilistic framework, going beyond the goals of the present study. At the same time, we are exploring the potential integration of deep learning–based approaches, such as autoencoders, to learn data-driven representations of allelic patterns directly from the data. Such models could complement the biologically grounded rules by capturing higher-order, non-linear dependencies that may not be fully described by frequency-based assumptions. This hybrid perspective may offer a promising direction for future methodological development.

A further methodological consideration concerns the assumption that a training population is available to learn the SOM representation. In practice, ASE datasets are never noise-free: they inherently contain technical variability, uneven coverage, mapping bias residuals, and biological stochasticity. Therefore, the “uncorrupted” training populations employed in our experiments should be interpreted as realistically noisy biological datasets, rather than idealized clean references. From a modeling perspective, the batch-training procedure of SOMs provides intrinsic robustness to moderate noise levels. Prototype vectors are iteratively updated as averages over multiple assigned samples and their neighbors, which reduces the influence of isolated perturbations and distributes local distortions across the map topology. This property makes SOMs particularly suitable for small-cohort genomic contexts, where explicit denoising or large reference panels may not be available. Nevertheless, systematically evaluating the impact of structured lack or corruption within the training population itself represents an important direction for future work. Such an analysis would require defining realistic generative models for ASE-specific noise (e.g., allele-specific dropout or regional coverage bias), reaching beyond the scope of the present methodological study.

Considering the shared representation between SOMs and k -means, we have to point that, in the analysis of grapevine dataset, both methods have been configured with 16 representative units. This empirical similarity is theoretically consistent and methodologically understandable, and can be interpreted as a consequence of three factors: *i)* both methods use 16 prototypes. This fixes the granularity of the partition and constrains both algorithms to explain the data with identical representational capacity; *ii)* because the SOM neighborhood radius shrinks during training, the final solution is largely determined by the tuning phase, where cooperative effects are minimal. In practice, this means that the final configuration is driven by quantization error minimization rather than topological ordering; *iii)* if the empirical data exhibit relatively compact clusters, approximately convex structure, and limited manifold curvature, then both learning algorithms naturally converge to similar Voronoi partitions. In such scenarios, the topological preservation mechanism of the SOM does not strongly alter the final centroids, leading to clustering assignments that closely match those of k -means. Although the similarity between SOM and k -means is theoretically justified, several methodological limitations should be acknowledged. First, using the same number of units ensures comparable representational capacity, but it does not ensure identical inductive biases. The SOM introduces a structural constraint (grid topology), whereas k -means does not. If the analysis does not explicitly evaluate topological properties (e.g., U-matrix, neighborhood consistency), the added value of SOM may remain underexplored. Second, both approaches are sensitive to initialization. Therefore, without multiple runs and stability analysis, apparent similarity might partly reflect initialization effects. Third, during the ordering phase, SOMs optimize a different effective objective due to the neighborhood term. If training duration is long enough, this phase may have limited impact on final weights. However, in shorter training regimes, differences could be more pronounced. Fourth, if the comparison relies primarily on cluster assignments or within-cluster variance, the evaluation implicitly favors k-means–type objectives. A more comprehensive comparison would include: topological error, quantization error, cluster stability metrics, external validation indices (if ground truth exists).

Finally, as further experiments, *i)* we will apply our approach to the entire genome of all tissues present in grapevine variants dataset to study *cis-regulatory* diversity at population level, *ii)* we will use it on the dataset developed in (Chen et al., 2016).

## 5 Conclusion

We developed a two-phase procedure to recover missing ASE data from RNA-seq experiments and to identify anomalous genomic regions. Even if we observed promising results, we acknowledge a number of issues ought to be addressed in future works. Firstly, a more relevant issue concerns error detection: in its current state, the procedure, simply looking at an expression vector $e_{\hat{p}}$ associated to a corrupted individual $\hat{p}$ , cannot distinguish whether a gene is homozygously (with no errors) or heterozygously (with one error) expressed; similarly, if $e_{\hat{p}}$ is 0 for all alleles of some gene g , the procedure cannot tell whether some error(s) occurred or g is simply not expressed in $\hat{p}$ . To address this, we may implement a prediction system, based on either the read-counts and frequencies of observed alleles or on external information. Moreover, we believe giving the end user only the most likely solution may be too restrictive: using statistics computed on training subpopulations, we may instead provide a ranked selection of different solutions. We also stress that a systematic benchmarking against dedicated CNV-calling or allelic-shift detection tools was not the primary objective of this study, given the different inference paradigms and target outputs. Nevertheless, evaluating the relationship between topology-based distortion signals and explicit dosage modeling frameworks represents an interesting direction for future work.

Concluding, we emphasize that the proposed approach can be adapted to solve related challenges in which data retrieval or imputation of missing data are needed. It could be very useful for preserving cases in a dataset instead of discarding them, mainly in situations where experiments are challenging and/or costly. A promising future direction involves coupling our framework with generative AI architectures designed to model the probabilistic structure of ASE. This integration could enhance biological realism by explicitly accounting for allelic variability and dispersion patterns while preserving the interpretable, population-level structure captured by the map. In this light, integrating topology-based clustering with biologically informed generative modeling may represent a promising avenue toward more realistic and structurally coherent reconstruction of allele-specific expression profiles in sparse genomic settings.

## Data Availability

Publicly available datasets were analyzed in this study. This data can be found here: 10.1038/s41467-021-27487-y.

## Footnotes

1 We anticipate that our dataset considers 17,000 features, i.e., possible expressed alleles, on a training population of less than 100 samples.

2 Henceforth, just the *signature* of p , for brevity

3

$\left({\hat{\alpha }}_{1},{\hat{\alpha }}_{2}\right)$ is the actual pair, which we aim to correctly identify after the reconstruction. Note that it may be ${\hat{\alpha }}_{1}={\hat{\alpha }}_{2}$ , if g is homozygously expressed.
